# Supplementary material for: Developing a comprehensive model of home-based formal care for elderly adults in Iran: A study protocol
Source: PLoS One. 2023 Aug 8;18(8):e0284462. doi: 10.1371/journal.pone.0284462 (PMC10409284; doi:10.1371/journal.pone.0284462)
Supplement: Attachment — Submitted filename: Response to Reviewers letter.docx [file pone.0284462.s002.docx]

December, 2022

Dear Bernadith Millamina
Straive Editorial Assistant

[PLOS ONE](https://www.google.com/url?sa=t&rct=j&q=&esrc=s&source=web&cd=&cad=rja&uact=8&ved=2ahUKEwifi-2g_9X7AhWng_0HHcv_DE0QFnoECCwQAQ&url=https%3A%2F%2Fjournals.plos.org%2Fplosone%2F&usg=AOvVaw1MOEO7pPI6Em5oSOFOaLMg)

Re: Submission ID PONE-D-22-23998: **Developing a Comprehensive Model of Home-Based Formal Care for Older Adults in Iran: A Study Protocol**

We want to thank you and the reviewers for the received comments, which provided us with the opportunity to improve the manuscript. We are pleased to inform you that the authors have had a detailed look at the manuscript and have addressed all comments, as much as possible, accordingly. Those changes are highlighted within the manuscript. Please see below, in blue, for a point-by-point response to the reviewers’ comments and concerns. All page numbers refer to the revised manuscript file with highlighted changes.

Yours truly,

Hossein Matlabi

Corresponding author

On behalf of the authors

Reviewers' comments:

Reviewer's Responses to Questions

**Comments to the Author**

1. Does the manuscript provide a valid rationale for the proposed study, with clearly identified and justified research questions?

The research question outlined is expected to address a valid academic problem or topic and contribute to the base of knowledge in the field.

**Response:** Thank you, required information is now added and highlighted in the introduction section (Pages 3 and 4, Lines 82-83; 87-93; 99-118; 124-127).

2. Is the protocol technically sound and planned in a manner that will lead to a meaningful outcome and allow testing the stated hypotheses?

The manuscript should describe the methods in sufficient detail to prevent undisclosed flexibility in the experimental procedure or analysis pipeline, including sufficient outcome-neutral conditions (e.g. necessary controls, absence of floor or ceiling effects) to test the proposed hypotheses and a statistical power analysis where applicable. As there may be aspects of the methodology and analysis which can only be refined once the work is undertaken, authors should outline potential assumptions and explicitly describe what aspects of the proposed analyses, if any, are exploratory.

**Response:** Thank you, required information, proposed hypotheses in the phase 1 and details of methodology is added and highlighted in the methods section (Page 5, Line 145-164; Page 6, line 165-196; Page 7, line 197- 228; Page 8, 229-246).

3. Is the methodology feasible and described in sufficient detail to allow the work to be replicable?


Descriptions of methods and materials in the protocol should be reported in sufficient detail for another researcher to reproduce all experiments and analyses. The protocol should describe the appropriate controls, sample size calculations, and replication needed to ensure that the data are robust and reproducible.

**Response:** Thank you, required details like study design, sampling, statistical analysis and other details were added and highlighted in the methods section (Page 5, Line 145-164; Page 6, line 165-196; Page 7, line 197- 228; Page 8, 229-246).

4. Have the authors described where all data underlying the findings will be made available when the study is complete?

The [PLOS Data policy](https://journals.plos.org/plosone/s/materials-and-software-sharing) requires authors to make all data underlying the findings described in their manuscript fully available without restriction, with rare exception, at the time of publication. The data should be provided as part of the manuscript or its supporting information, or deposited to a public repository. For example, in addition to summary statistics, the data points behind means, medians and variance measures should be available. If there are restrictions on publicly sharing data—e.g. participant privacy or use of data from a third party—those must be specified.

**Response:** No datasets were generated or analysed during the current study. All relevant data from this study will be made available upon study completion.

5. Is the manuscript presented in an intelligible fashion and written in standard English?

PLOS ONE does not copyedit accepted manuscripts, so the language in submitted articles must be clear, correct, and unambiguous. Any typographical or grammatical errors should be corrected at revision, so please note any specific errors here.

**Response**: There is no comment for this case.

6. Review Comments to the Author

Please use the space provided to explain your answers to the questions above and, if applicable, provide comments about issues authors must address before this protocol can be accepted for publication. You may also include additional comments for the author, including concerns about research or publication ethics.

You may also provide optional suggestions and comments to authors that they might find helpful in planning their study.

(Please upload your review as an attachment if it exceeds 20,000 characters)

Reviewer #1: Extremely important topic and very interesting approach to a potential strategy. Suggestions:
- Revising the definition of informal caregivers on the introductions, because it seems it is a little mixed with the next definitions of formal caregivers ("...or people who are privately employed by the older adults or their family members..." - if they are employed, they are formal caregivers).

**Response:** Thank you, we revised the whole introduction according to the main goal of the study. we also revised the informal caregiver definition according to the latest evidence and highlighted in the introduction section (Pages 3 and 4, Lines 82-83; 87-93; 99-118; 124-127).

- Do not use the term "elderly" - according to the latest WHO Report to Combat Ageism, there is a negative connotation in this term, and the correct is older people/older adult/older person

**Response:** Thank you, we replaced elderly with older adults and older person in whole the manuscript.

- The introduction should also highlight the importance of preventing care needs, favoring actions to promote functional ability AND having a more structured long-term care/home care for those individuals that will need such support

**Response:** Thank you, we addressed all of your recommendations in the introduction section and highlighted in the introduction section (Page 4, Line 103-108).

- Better definition on the "home care" - when explained on the introduction, it seems is focused on "formal care" - provided by health providers. But further in the introduction the text relates also to informal caregivers and the needs related to training and support. This should be clarified.

**Response:** Thank you, we revised the introduction section and explained that in order to reduce the burden of informal care, to reduce hospitalization costs, and due to the change in family structure and women's employment, developed countries have established a formal home care system. This system is also needed in Iran. Therefore, the presented model is also for formal care at home in which informal caregivers are also involved.

- Methods (Phase 1 Step 1): I believe the ratio from dependent to independent is very broad, because the level of dependency varies too much, and this implies the training caregivers should receive. I suggest considering support on types of activities (basic x instrumental - as it is being collected from the instruments) and from there have different levels of dependency. Explain why 636 older adults as sample (was sample size calculated, how?). Also, it is confusing that it said sample will be OA, but later said the interview can be done with the caregivers. Both individuals might have very different perspectives and should be explained when/why caregiver could be a proxy. For example: The cognitive disorder is an exclusion criteria (I imagine for OA). In this case, caregivers couldn't be a proxy?
When the content and face validity would be conducted?

**Response:** We explained the details of sampling in phase 1 step 1. According to your suggestion, we added that the study samples will be older people, but if the older person has cognitive impairment, the questions will be asked from the person closest to the older person (Page 5 and 6, line 156-176).

After designing the instrument, the content and face validity would be conducted (Page6, line 187-190).

- Methods (Phase 1 Step 2): Saturation of data is not usually dependent on the time (revise the sentence says it would take approximately 6 months - this is the expected timeline for the researchers to achieve saturation from the interviews conducted). Include the key informants as part of the sample in the beginning of the section. I just understood they would be part of the interviews after I checked the table. On the questions, that has to be included a question for paid caregivers on how they feel about providing care (positive and negative feelings). It is a little confusing that it seems the first step includes older adults receiving informal care and the second only formal home care. Why?

**Response**: Thank you, we added required information about data saturation and guide questions in the qualitative step (Page 7 and 8, Lines 200-246).

The first step aims to assess whether older adults receive informal care or paid care? Is the care related to assistance with activities of daily living (ADL, IADL) or health-related care, and who provides this care (family or paid caregiver)? In the next step, we will explore the challenges of receiving paid care, whether receiving this type of care is structured or unstructured? After obtaining the necessary information, we can design the appropriate home care model for older adults.

- Methods (Phase 2 Step 1): Long-term care in general or formal home care? The question must be very specific (in objectives it is stated to understand "home-based long-term care for older adults in different countries".

**Response**: We have corrected this statement (page 8, line 259-260).

- Discussion and overall objectives: I believe it is a very important strategy but as stated in the introduction, most care is provided by informal caregivers and here is a model for formal care. The discussion should address how this model will be helpful to be supportive for those there already receive formal care but also maybe more accessible for all? Or should other recommendations be provided for the informal?

**Response**: As explained in the answer to the previous comments, we have modified both the discussion and the introduction (Discussion section, page 12, line 370-382).

Reviewer #2: The design and implementation of long-term care services within ageing societies is crucial. This study protocol intends to provide and stepwise approach to build on an appropriate home care model. Nevertheless, in this protocol there are some key issues that prevents to understand how the authors will answer their research question, which includes:
1. Which conceptual framework will be used to define functional ability and how disability will be defined in this research?

**Response**: Thanks for informative comment. We explained and highlighted the conceptual framework used for disability in the introduction and explained how disability will be defined in this study (Page 5, line 145-155).

2. Considering care dependency also affects persons from all ages, why only including participants 60+? A wider approach is recommended

**Response:** Thank you. You have a holistic view, but the target group of this study is for older adults and therefore we wouldn't include other age groups in the study.

3. How the needs of people living with dementia and their careers will be assessed?

**Response:** Thank you, in step 2 phase 1, caregivers of older people with dementia who receive paid care, will be included in the interview. We will address their challenges and needs.

In step 1 phase 1, if older adults have a cognitive disorder, the person closest to the older person will be asked questions as the representative.

4. How the perceptions of care of those that currently do not have access to care will be capture?

**Response**: Thank you, the main aim of this study is to design a comprehensive model of home-based formal care. Therefore, to achieve the aim of the step, we want to discover the experiences and challenges faced by people who receive care from paid caregivers, so people who have enough experience in receiving this type of care should be included in the study.
