# Supplementary material for: Developing a comprehensive model of home-based formal care for elderly adults in Iran: A study protocol
Source: PLoS One. 2023 Aug 8;18(8):e0284462. doi: 10.1371/journal.pone.0284462 (PMC10409284; doi:10.1371/journal.pone.0284462)
Supplement: Attachment — Submitted filename: PONE.docx [file pone.0284462.s003.docx]

Developing a Comprehensive Model of Home-Based Formal Care for Older Adults in Iran: A Study Protocol

Reviewer comments:

1. Please check the grammar errors in the manuscript. For example: The title: it should be Elderly adults, not older adults (replace it in all manuscript)

In abstract: grammar errors: “few studies are in the home care area in developing countries.” It should be: few studies are conducted to evaluate home-based care model in the developing countries.

2. In methodology: phase 2: what is the relevance of using the data base for searching while your study is not a systematic review.
3. In abstract: the last paragraph is a conclusion not a discussion. Furthermore, there is no result paragraph in the abstract.
4. In the objectives: it is another study to make a review of the structure of home care for older adults. All data related to this part should be shredded and you may use it to prepare a separated systematic review for example.
5. The validation of the model is part of the development and not a separated objective.
6. The background is too long, it should be summarized to important and related studies.
7. The study design is confused and not clear. It should be rebuilt. It is a paper and not a thesis.
8. No result, no conclusion in the manuscript.
9. The discussion is very plain in comparison with the methodology and the supposed results.
