# Supplementary material for: Developing a comprehensive model of home-based formal care for elderly adults in Iran: A study protocol
Source: PLoS One. 2023 Aug 8;18(8):e0284462. doi: 10.1371/journal.pone.0284462 (PMC10409284; doi:10.1371/journal.pone.0284462)
Supplement: Attachment — Submitted filename: Response to Reviewers.docx [file pone.0284462.s004.docx]

**Journal of [PLOS ONE](https://journals.plos.org/plosone/)**

**Dear Editor**

**Subject:** Developing a Comprehensive Model of Home-Based Formal Care for Elderly Adults in Iran: A Study Protocol

Submission of revised Manuscript Ref: PONE-D-22-33262

Thank you for your email dated 21 Feb 2023 enclosing the editor and reviewers’ comments. We have carefully reviewed the comments and have revised the manuscript accordingly. Our responses are given in a point-by-point manner below. Changes to the manuscript are shown in the track changes.

Sincerely,

Hossein Matlabi

Tabriz University of Medical Sciences- Tabriz – Iran

Comments from the Editors and Reviewers:

| **Editor’s comments** | |
| --- | --- |
| comment | Response |
| Provide elaboration on the justification of using content analysis in Phase 1, Step 2 | Thank you for this comment. We explained the reason for using a content analysis approach to analyze qualitative data (page 12, line 271-277). |
| Justify the exclusion of experimental studies in the scoping review protocol | Thanks for your comment. The philosophy of conducting experimental studies is to test the effect of independent variable(s) on dependent variable(s) and considering that the aim of this scoping review is to achieve the structure of home care in different countries, therefore, experimental studies cannot answer the research question. |
| Please ensure that your manuscript meets PLOS ONE's style requirements, including those for file naming. The PLOS ONE style templates. | Thank you. We revised the article according to PLOS ONE's style requirements. |
| . Your ethics statement should only appear in the Methods section of your manuscript. If your ethics statement is written in any section besides the Methods, please delete it from any other section. | We removed the ethics statement from the other sections and added it to the method section (page 7, lines 154-159) |
| Please include a copy of Table 3 which you refer to in your text on page 9. | Thank you. We have not mentioned Table 3 in the text because Table 3 does not exist. |
| Please review your reference list to ensure that it is complete and correct. If you have cited papers that have been retracted, please include the rationale for doing so in the manuscript text, or remove these references and replace them with relevant current references. Any changes to the reference list should be mentioned in the rebuttal letter that accompanies your revised manuscript. If you need to cite a retracted article, indicate the article’s retracted status in the References list and also include a citation and full reference for the retraction notice. | Thank you for your comment. We reviewed the list of references for accuracy. Because this article has been changed compared to the first version, therefore, we used the references related to the updated text. |
| **Reviewer #1** | |
| Have the authors described where all data underlying the findings will be made available when the study is complete? NO | No datasets were generated or analysed during the current study. All relevant data from this study will be made available upon study completion. |
| What are the specific countries that you studied in order to extract the main components of organized care? | Thank you for your valuable comment. We will not limit the selection of countries because otherwise we may lose the information of some countries. With a systematic search, any country that has information on the structure of home care will be found, and we will include the information from these countries in our study. However, not all countries have a structured home care system for older adults, and this type of structure is more common in advanced countries such as Germany, Japan, South Korea, Denmark, Sweden, the United States of America, England, Switzerland, and similar countries. We added the justifications to the main text. (page 13, lines 309-313) |
| Can you provide more information about the Delphi study, specifically the number of rounds used and the criteria for selecting experts? | Thank you for this valuable comment. We provided and highlighted in the text additional information about the number of Delphi rounds and why these rounds should be. (page 16, lines 369-376)  Due to the fact that experts in the geriatric field will be included in the Delphi study, therefore, the inclusion criteria for the study’s participants in the Delphi study will be the same as the criteria in the expert panel. We have included this sentence in the text repeated (page 16, lines 381- 383, 386-388). |
| Can you describe the data extraction form that will be used in phase 3 and explain its purpose? | Thank you for your comment. The purpose of designing this form is to create a regular schedule and prevent data missing. This form can help to classify and organize the data obtained from different documents in a structured and systematic way and give a comprehensive view to the reader. The information on title, type (Law, policy, regulation or other types of documents), the principle, paragraph or article of the document, publication date and place, stakeholders and content of the document will be included in the form. We added this information in the text (page 15, lines 340- 346). |
| How will you ensure that the participants in the Delphi study are representative of the population of older adults living in Iran? | Thank you for closely attention to the matter. Due to the fact that we will include experts in the Delphi study who have sufficient work experience and knowledge in the geriatrics field, therefore, because these experts are closely involved with the older adults’ issues, they can be a proper representative of the population of older adults living in Iran. |
| Can you explain how the sample size was calculated and why the stratified-cluster sampling method was chosen? | Thank you for your comment. The Cochran’s Sample Size Formula was used to calculate the number of people who will enter the study. We have already included the details of the sample size calculation in the text. We highlighted this information.  The study used stratified- cluster sampling design. Simple random sampling across the Tabriz city could have been utilized to recruit older adults. However, using the simple random sampling, a large enough older people were needed to be representative sample. The population of Tabriz is geographically diverse. Therefore, random sampling of older adults across the city would have been impractical and expensive. Stratified- cluster sampling is effective in reducing sampling error [1, 2]. A stratified cluster sampling approach was therefore used to ensure the selected sample was representative of the city. We added this justification to the text (page 8, lines 177-190; 201-206). |
| In order to assess the proposed model from a financial perspective, I also urge you to think about integrating a cost-benefit analysis or cost-effectiveness analysis in your research. Examining the related financial matters, potential funding sources, and healthcare system savings for the proposed model's adoption could be helpful as well. | Thank you for this valuable comment. In this model, effective resource allocation mechanisms will be clarified. For this purpose, using the experiences of advanced countries as well as the results of interviews with experts, financial resources will be determined according to the context of Iran. We added some information about financing in the discussion section (page19, lines 443-450). |
| **Reviewer #2** | |
| Please check the grammar errors in the manuscript. For example: The title: it should be Elderly adults, not older adults (replace it in all manuscript)  In abstract: grammar errors: “few studies are in the home care area in developing countries.” It should be: few studies are conducted to evaluate home-based care model in the developing countries. | Thank you for this comment. We checked the grammatical errors and corrected them. We replaced elderly adults with older adults in all manuscript. |
| In methodology: phase 2: what is the relevance of using the data base for searching while your study is not a systematic review. | Thank you for this comment. It has been emphasized that one of the main methods for finding the answer to a question in the scoping review is a comprehensive search among a set of databases and one type of scoping review is a systematic scoping review in which reliable databases should be used for searching [3]. In addition, reliable websites and gray literature can also be helpful and useful [4]. |
| In abstract: the last paragraph is a conclusion not a discussion. Furthermore, there is no result paragraph in the abstract. | Thanks for your insights and guidance. We changed the discussion section in the abstract.  Because this article is a protocol study and the results have not been obtained, therefore the abstract and the main text do not have the results section. |
| In the objectives: it is another study to make a review of the structure of home care for older adults. All data related to this part should be shredded and you may use it to prepare a separated systematic review for example. | Thank you for your comment. In order to design a home care model, we need to extract different aspects of home care in a study and use its results in the development of model. Considering the nature of the scoping review, which is preferable to the systematic review in clarifying the key concepts and dimensions of a subject, we chose scoping review [5]. |
| The validation of the model is part of the development and not a separated objective. | Thanks for your comment. We added validation of the model with model development and removed Phase 4. (Page 15, line 335) |
| The background is too long, it should be summarized to important and related studies. | Thank you. We removed additional information from the introduction section. |
| The study design is confused and not clear. It should be rebuilt. It is a paper and not a thesis. | Thanks for your comment. In order to provide a comprehensive model of home care for the Iranian elderly, it was necessary to design a comprehensive and well-considered method. The use of mixed-method, including a quantitative survey and qualitative interviews, clarifies all issues related to home-based care in Iran. In the following, it is appropriate to design a scoping review to determine the domains of home care in other countries, the triangulation approach to develop a model for the Iranian context, and a Delphi study to evaluate the model's feasibility and reliability. |
| No result, no conclusion in the manuscript. | Thank you for useful comment. We added conclusion section to the manuscript (page 20, line 461-469), but since this is a protocol study, there are currently no results to report in the article. |
| The discussion is very plain in comparison with the methodology and the supposed results. | Thank you. We removed additional information from the introduction section and improved the discussion section by referring to related studies (page 18, lines 409-415, 423-428, 430-433; page 19, lines 436-439, 443-450). |

1.	Pu X, Gao G, Fan Y, Wang M. Parameter estimation in stratified cluster sampling under randomized response models for sensitive question survey. Plos one. 2016;11(2):e0148267.

2.	Sedgwick P. Stratified cluster sampling. Bmj. 2013;347.

3.	Peters MD, Godfrey CM, Khalil H, McInerney P, Parker D, Soares CB. Guidance for conducting systematic scoping reviews. JBI Evidence Implementation. 2015;13(3):141-6.

4.	Munn Z, Peters MD, Stern C, Tufanaru C, McArthur A, Aromataris E. Systematic review or scoping review? Guidance for authors when choosing between a systematic or scoping review approach. BMC medical research methodology. 2018;18:1-7.

5.	Tricco AC, Lillie E, Zarin W, O’brien K, Colquhoun H, Kastner M, et al. A scoping review on the conduct and reporting of scoping reviews. BMC medical research methodology. 2016;16:1-10.
